# Ultraviolet Fluorescence Imaging of Fingerprints

**DOI:** 10.1100/tsw.2006.143

**Published:** 2006-06-21

**Authors:** Naoki Saitoh, Norimitsu Akiba

**Affiliations:** Second Forensic Science Division, National Research Institute of Police Science, 6-3-1 Kashiwanoha, Kashiwa, Chiba 277-0882, Japan

**Keywords:** fingerprint, ultraviolet fluorescence image, Nd-YAG laser, time-resolved imaging, ultraviolet absorption image

## Abstract

We studied fluorescence imaging of fingerprints on a high-grade white paper in the deep ultraviolet (UV) region with a nanosecond-pulsed Nd-YAG laser system that consists of a tunable laser and a cooled CCD camera.Clear fluorescence images were obtained by time-resolved imaging with a 255- to 425-nm band-pass filter, which cuts off strong fluorescence of papers. Although fluorescence can be imaged with any excitation wavelength between 220 and 290 nm, 230 and 280 nm are the best in terms of image quality. However, the damage due to laser illumination was smaller for 266-nm excitation than 230- or 280-nm excitation.Absorption images of latent fingerprints on a high-grade white paper are also obtained with our imaging system using 215- to 280-nm laser light. Shorter wavelengths produce better images and the best image was obtained with 215 nm. Absorption images are also degraded slightly by laser illumination, but their damage is smaller than that of fluorescence images.

## INTRODUCTION

In this paper, we studied the imaging method of latent fingerprints on white papers using ultraviolet (UV) fluorescence. About fingerprint imaging using UV rays, Ohki[1] first took a UV absorption photograph using a 365- and 253-nm filter with a xenon-mercury lamp. Saitoh and Arai first reported the method using a UV television system in 1972[2]. They used a filter that was transparent for 274 ± 15 nm with a xenon lamp and took absorption images of fingerprints on plastics. For imaging fingerprints using UV fluorescence, Bramble et al.[3] reported the method using 266-nm light from a Nd-YAG laser.

In a previous paper[4], we showed that fluorescence spectra of fingerprints have two main peaks, at 330 nm (peak A) and at 440 nm (peak B), when excited with 266-nm laser light from a Nd-YAG laser. The 330-nm peak reduced its intensity as laser illumination proceeded. On the other hand, the 440-nm peak was not prominent at first or just after pressed, but its intensity increased slightly as time elapsed.

At first, we measured the fluorescence spectra of high-grade (HG) white papers and found that they emit strong fluorescence in the same wavelength range of peak B. Therefore, we conducted fluorescence imaging of latent fingerprints on white papers using peak A. We will show time-resolved fluorescence images of latent fingerprints on HG white papers. Time variation due to illumination of laser light is also shown. In the last section, we will refer to UV absorption images of fingerprints.

## EXPERIMENT

The block diagram of our system is depicted in Fig. 1. This system is the same as described in our previous paper[4]. The system consists of a pulsed Nd-YAG laser (Continuum, Powerlite Precision 8010), OPO (Optical Parametric Oscillator) + Doubler (Continuum Panther EXST-1), a cooled CCD camera with an image intensifier (ICCD camera) (Roper Scientific, PI-MAX 1K-RB-FG-43), and a spectrometer (Acton Research Corporation, SpectraPro-300i). This laser is a tunable laser that generates light with broad wavelength from 215–2550 nm. The frequency of laser pulse is 10 Hz and the width of the individual pulse is about 5 nsec. The energy of the laser pulse depends on the wavelength and is about 3~4 mJ for 220- to 300-nm wavelength on average. Laser pulses were irradiated onto samples after expanding the beam with a beam expander or a holographic diffuser. With this system, time-resolved fluorescence images can be measured using the fast-gated image intensifier. The image intensifier can be gated faster than 2 nsec. The gating control of exposure time of the ICCD camera or delay time of observation is done by a PTG (programming timing generator) and a personal computer. The gate width is set to 10 μsec. The gate width means the exposure time to the incident laser light.

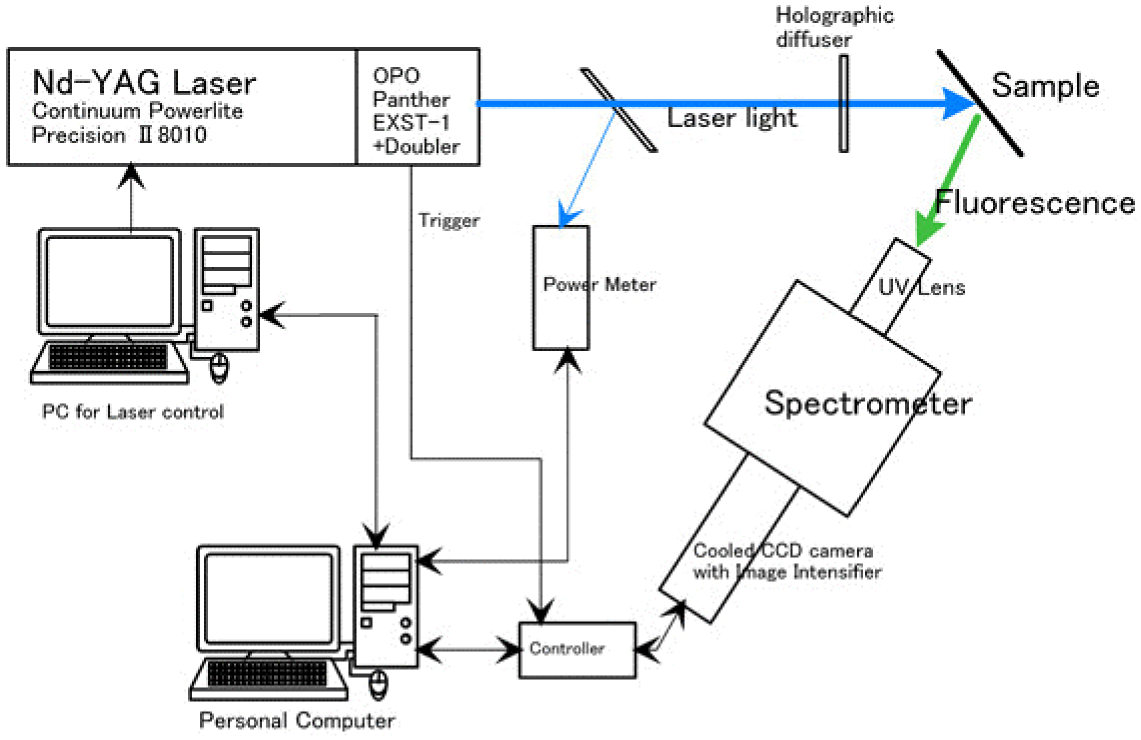

**FIGURE 1.** Block diagram of the time-resolving fluorescence imaging and spectroscopy system.

A fingerprint was pressed on a HG white paper that is 64 g/$m^2$ in weight. “High-grade” means that lignin is 100% removed from pulp. Before pressing the fingerprint, fingers were washed with soap and dried in the air. Then the fingerprint was pressed after wiping the forehead or nose. Fluorescence was collected with a UV lens and put into the spectrometer that has a grating monochromator with 300 g/mm.

However, for imaging, the monochromator was not used. Actually with this system, fluorescence images can be obtained by setting the center wavelength of the monochromator to 0 nm and removing a slit.

CCD of our system has 1024 × 1024 pixels of 13- × 13-μm pixel size. Obtained images were digitized in 16 bit. In order to suppress noise, CCD was cooled to –20°C. In addition, background noise image can be subtracted at each measurement. In the case of measurement of fingerprint fluorescence, it is necessary to accumulate many measurements because fluorescence intensity is weak. Since the laser-pulse frequency is 10 Hz and the gate width is 10 μsec, the total observation time becomes 0.1 msec/sec. The experiment was done in a room and all images were obtained under illumination of a fluorescent lamp.

In each imaging, 1000 observations were accumulated. Since the image brightness became low as the delay time increased, the contrast of each image was enhanced using Adobe Photoshop to show details clearly.

## IMAGING UV FLUORESCENCE OF FINGERPRINT

For imaging UV fluorescence of fingerprints, it is important to discriminate between fluorescence of fingerprints and that of background materials. Background materials such as papers, clothes, etc. are often added with fluorescent whitening agents that absorb UV light and emit fluorescence of 400- to 600-nm wavelength, which is in the same wavelength region of peak B fluorescence of fingerprints. In Fig. 2, time-resolved fluorescence spectra of a HG white paper are shown. These spectra were obtained with a 10- to 40-nsec delay from the laser-pulse excitation. The fluorescence ranges from 400 to over 500 nm, and overlaps with the wavelength range of peak B. The fluorescence lifetime was estimated from the figure as approximately 1.7 nsec.

Thus, since HG white papers emit strong fluorescence at above 400 nm, it is important to suppress paper fluorescence. There are two methods: one is to filter out the paper fluorescence and the other is the time-resolved imaging. At first, we conducted imaging of latent fingerprints on a HG white paper without a filter using 266-nm laser light. In this case, time-resolved imaging was needed, but clear fingerprint images were not obtained without a filter that cuts off the paper fluorescence. In Fig. 3, time-resolved images of latent fingerprints on HG white papers are shown for 10-, 50-, and 100-nsec delays. For all images in Fig. 3, ridge patterns are observed, but not clear. Next, the fingerprint was imaged with a UV transparent band-pass filter HOYA U-340. This filter is transparent for 255–425 nm. Therefore, the fluorescence of papers and peak B fluorescence are filtered out. Results are shown in Fig. 4 for 10-, 50-, and 100-nsec delays. For less than a 50-nsec delay, clear ridge structures were observed, but for a 100-nsec delay, clear images were not obtained due to low S/N ratio.

### Dependence on the Excitation Wavelength

Bramble measured the UV fluorescence of fingerprints using a fluorescence spectrophotometer[5] and found that fluorescence intensity becomes strongest when excited with a 280-nm wavelength. We have also measured UV excitation spectra of fingerprints[6]. According to the obtained excitation spectra, the fluorescence intensity of fingerprints becomes maximum at 230 and 280 nm. In Fig. 5, fluorescence images are shown for various excitation wavelengths. Detailed ridge structures can be observed for 220- to 290-nm excitation. As expected from the excitation spectra, the images obtained with 230- and 280-nm excitations show the best image quality.

For 215-nm excitation, fluorescence was weak and ridge structures were not observed. For more than 290-nm excitation, ridge structures are not recognized clearly. Thus, by measurements with a specific band-pass filter and the time-resolved imaging with the appropriate delay time, it is recognized that latent fingerprints on a HG white paper can be visualized.

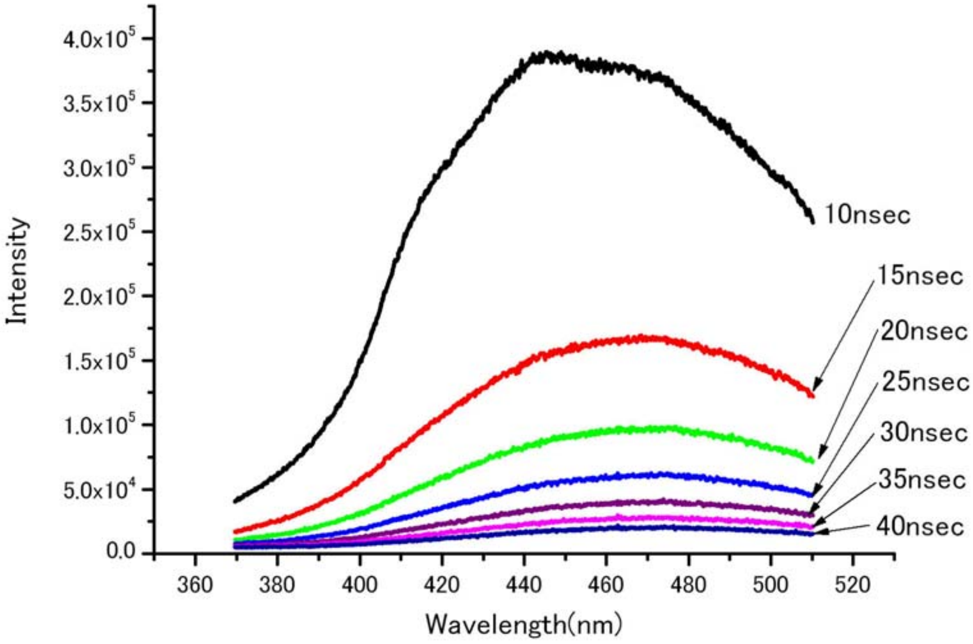

**FIGURE 2.** Time-resolved fluorescence spectra of a HG white paper.

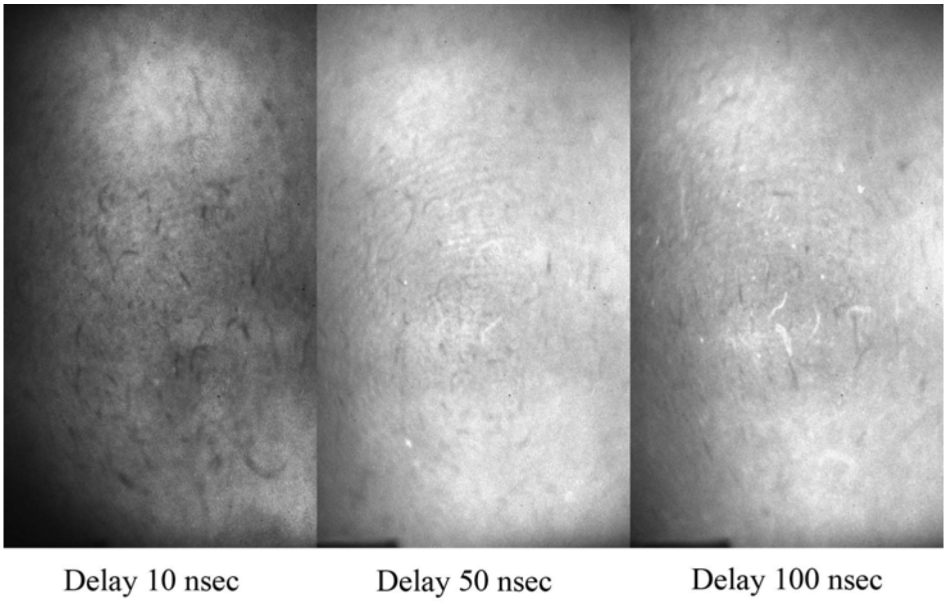

**FIGURE 3.** Time-resolved fluorescence images of latent fingerprints on a HG whitepaper without a filter excited with 266 nm.

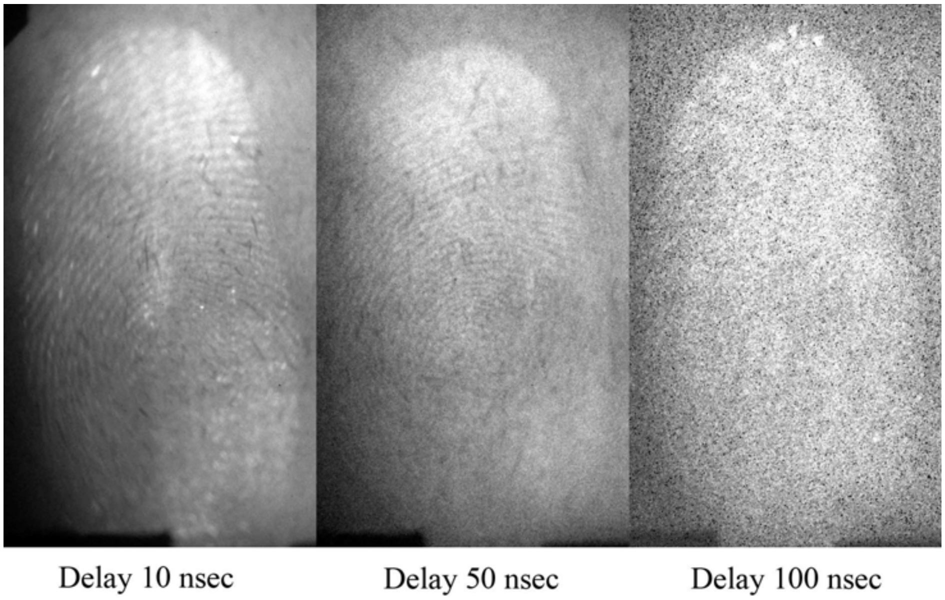

**FIGURE 4.** Time-resolved fluorescence images of latent fingerprints on a HG white paper with a band-pass filter excited with 266 nm.

## VARIATION OF FLUORESCENCE IMAGE OF FINGERPRINT BY LASER ILLUMINATION

As reported in a previous paper[4], peak A fluorescence of fingerprints reduces its intensity as laser-light illumination proceeds. Therefore, as pointed out by Bramble[5], the contrast of fluorescence image becomes lower as illumination time increases. We studied the influence of laser illumination on fingerprint fluorescence images and found that the effect by laser illumination depends on the excitation wavelength. In Fig. 6, fluorescence images are shown for various illumination times of laser light. The excitation wavelengths are 230, 266, and 280 nm. It can be clearly seen that the image contrast becomes lower as illumination time exceeds 20 min, but there is a difference in the speed of degradation among these three. In the case of 266-nm excitation, the ridge structures can be recognized even after 3 h of illumination, but in the case of 230- and 280-nm excitations, the fluorescence intensity becomes low very fast and it becomes difficult to recognize detailed ridge structures after 20 min. This means that 230- and 280-nm laser lights damage the fingerprint fluorescence faster than 266-nm laser light.

## ABSORPTION IMAGE OF FINGERPRINT

Ohki[1] studied UV absorption and fluorescence of human epidermal secretion and detected the absorption maximum at 277 nm that corresponds to urocanic acid. Ohki examined in detail the absorption property of human epidermal secretion that was extracted using ethanol-water from gauze, but he did not measure fingerprints directly. He also took UV absorption images of fingerprints on polyvinyl chloride resin and papers using 253- and 365-nm filters.

In our system, absorption images can be measured by setting the delay time as 0 nsec. Results are shown in Fig. 7. In Fig. 7, absorption images are shown for various excitation wavelengths. The quality of absorption images depends on the excitation wavelength. A better result was obtained as the excitation wavelength became short, between 215 and 350 nm. That is, in our experiments, the best absorption image was obtained with 215-nm absorption. It might be possible to obtain better images using a shorter wavelength than 215 nm. When the wavelength exceeds 260 nm, clear absorption images cannot be obtained.

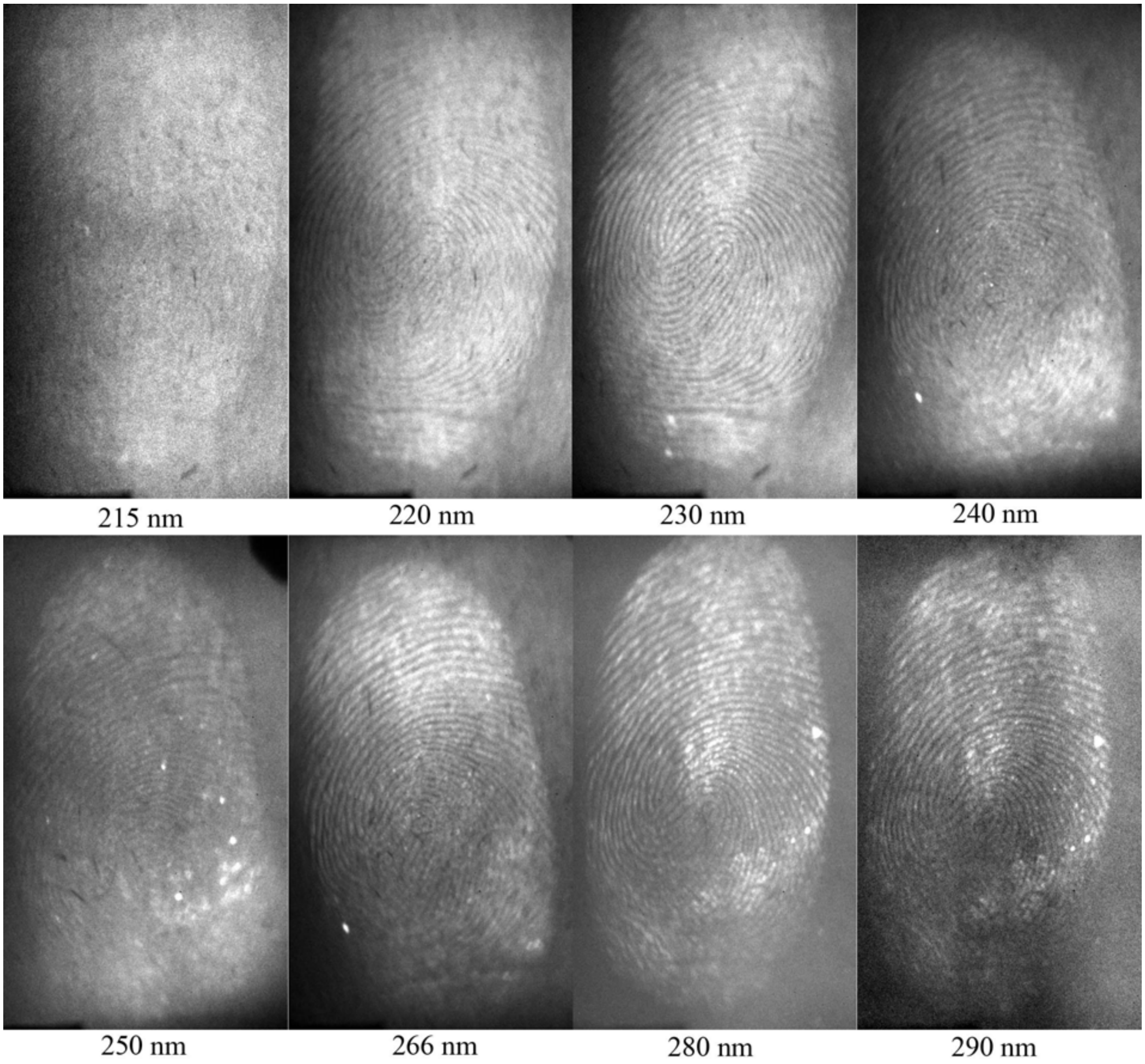

**FIGURE 5.** Fluorescence images of latent fingerprints on a HG white paper excited with various wavelengths.

The quality of absorption images is degraded by laser-light illumination like fluorescence images, but its speed of degradation is slower than that of fluorescence images. In Fig. 8, results of time variation of absorption images with 215-nm excitation are shown for a fresh fingerprint (0 min), after 3 h of illumination, and after being kept under a fluorescent lamp for 7 days without laser illumination. The image of ridge structures is slightly degraded by this process and even after 3 h, ridge structures remain clear. In addition, the degradation by laser illumination is so small that ridge structures can be recognized after 7 days.

## DISCUSSION

We studied the visualization of fingerprints on HG white papers with a nanosecond-pulsed laser and an image-intensified cooled CCD system. Fluorescent additives in papers emit strong fluorescence between 400–600 nm. Since the lifetime for peak B of fingerprints is approximately 6.2 nsec[1] and that of a HG white paper is approximately 1.7 nsec, the ratio of the fluorescence intensity of a fingerprint to that of a HG white paper is given as follows:

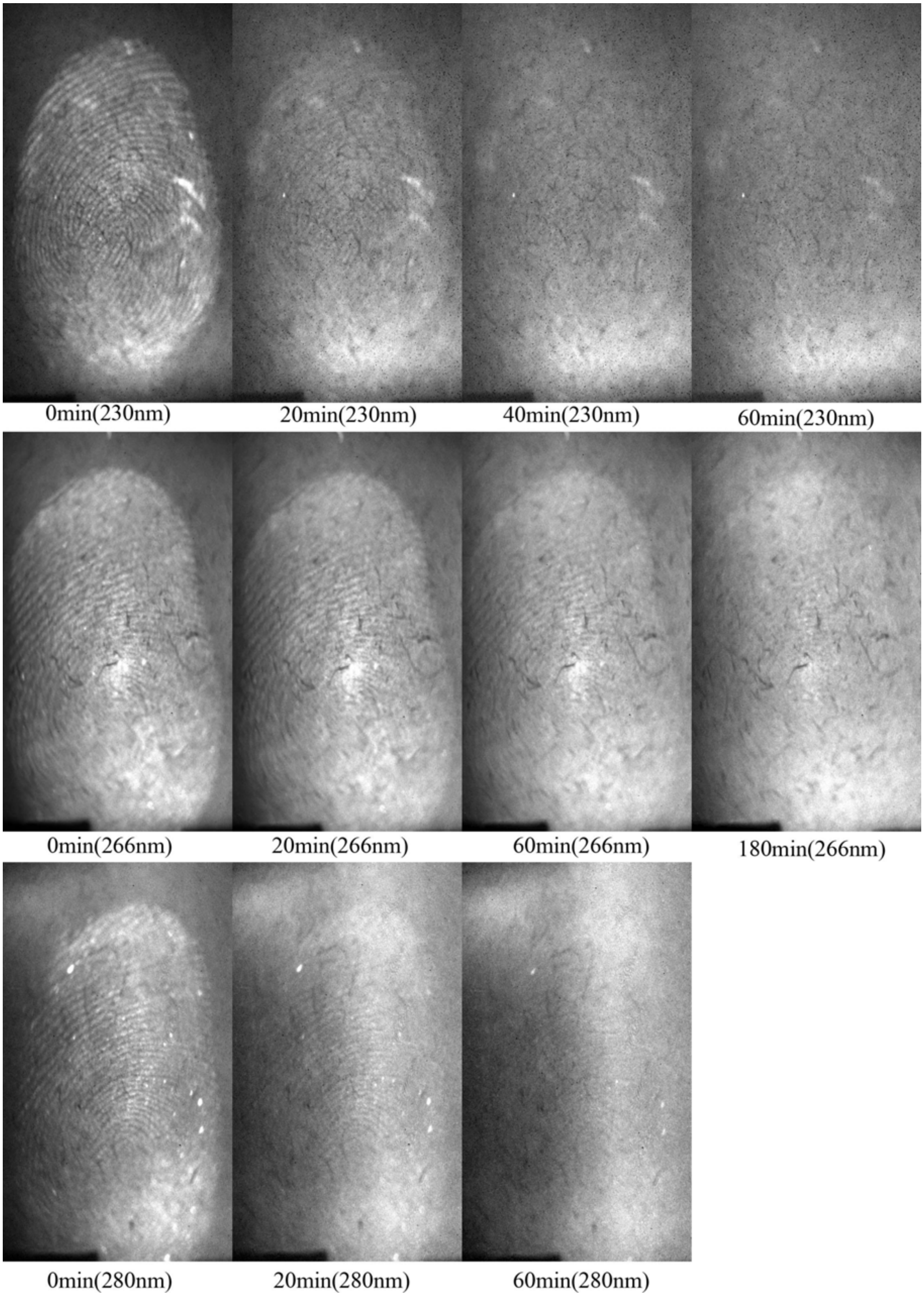

**FIGURE 6.** Time variation of fluorescence images of fingerprints on a HG white paper.

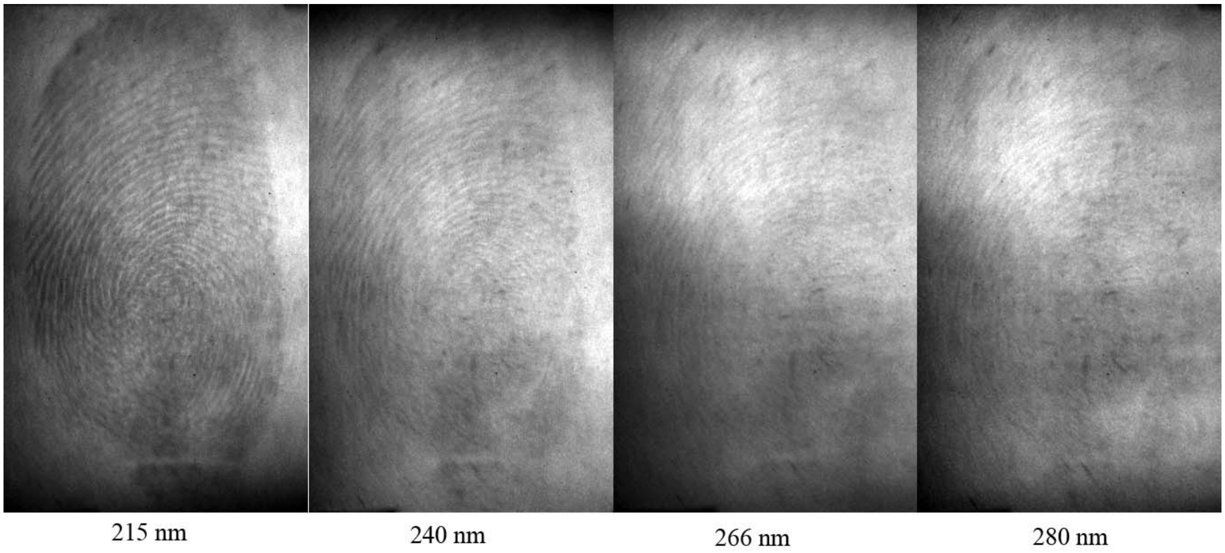

**FIGURE 7.** Absorption images of latent fingerprints on a HG white paper with various wavelengths.

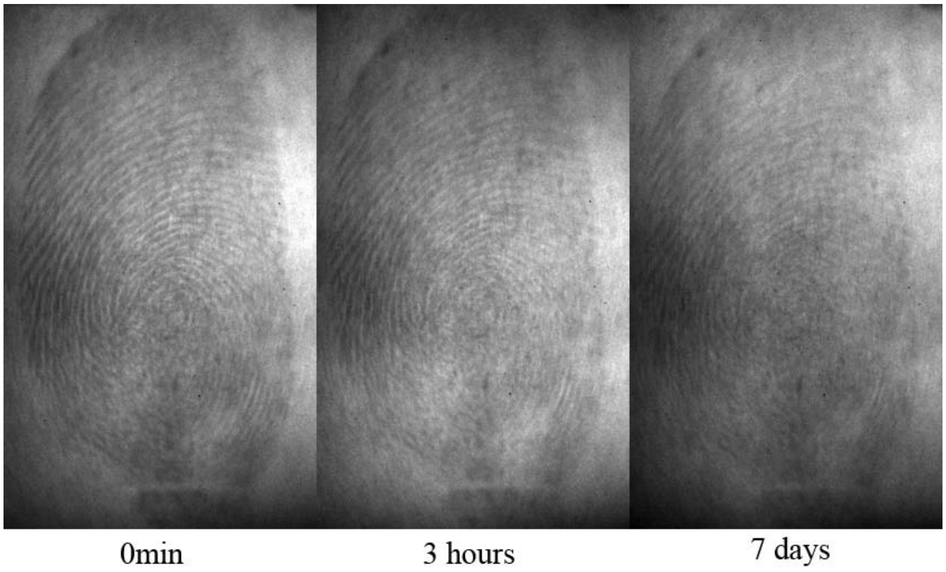

**FIGURE 8.** Time variation of absorption images of latent fingerprints on a HG white paper.

$$R = \frac{I_f}{I_p} = \frac{I_{f0} e^{-\frac{t}{\tau_f}}}{I_{p0} e^{-\frac{t}{\tau_p}}} = \frac{I_{f0}}{I_{p0}} e^{\frac{\tau_f - \tau_p}{\tau_p \tau_f} t} = R_0 e^{0.427t}, R_0 = \frac{I_{f0}}{I_{p0}},$$

where $I_f$ and $I_p$ represent the fluorescence intensity of a fingerprint and a HG white paper, respectively, and $I_{f0}$ and $I_{p0}$ are their initial fluorescence intensity. $\tau_f$ and $\tau_p$ represent the lifetime of a fingerprint and a HG white paper, respectively. $R_0$, the initial intensity ratio, was measured as the order of $10^{-4}$. Therefore, in order to achieve $R > 1$, it is necessary that $t > 30$ nsec. In our fluorescence imaging without a filter, S/N

ratio was improved gradually as the increase of the delay time (Fig. 3). However, its image quality is not clear enough for recognizing ridge structures even for more than a 50-nsec delay. This may be caused by the paper fluorescence with a longer lifetime. Therefore, the best way to image UV fluorescence of fingerprints on a HG white paper is to use a band-pass filter (Fig. 4). In such a case, the time-resolved imaging is not essential. However, by the time-resolved imaging, fluorescence of fingerprints can be visualized even under ambient lights.

In this paper, we also studied the absorption images of fingerprints. As a result, clear images were obtained using 215-nm laser light. Although absorption images can be obtained with 240-nm laser light, a shorter wavelength produces a better image. In the case of absorption imaging, the damage by laser-light illumination is not so large that even with the 3-h illumination of 215-nm laser light, a clear image can be obtained. The absorption image contrast becomes gradually low by being left under a fluorescent lamp.

Fingerprints change their spectral profile by laser illumination, that is, the peak position of their primary spectrum component moves from 330 nm (peak A) to 460 nm (peak B). Therefore, the contrast of fluorescence images using peak A becomes low as laser illumination proceeds. This suggests that the laser illumination duration time should not be large. Since it takes only a few minutes to obtain fluorescence images of fingerprints with our system, the effect of laser illumination for imaging fingerprint is small.

## ACKNOWLEDGMENTS

The authors would like to thank Dr. Shigeki Takeuchi of the Criminal Investigative Laboratory of Gifu Prefectural Police Headquarters for his helpful discussions and Dr. Kenro Kuroki of the National Research Institute of Police Science for his assistance in the experiment.
